# Researching male circumcision for HIV prevention in Papua New Guinea: a process that incorporates science, faith and culture

**DOI:** 10.1186/1478-4505-11-44

**Published:** 2013-11-13

**Authors:** Rachael Tommbe, David J MacLaren, Michelle L Redman-MacLaren, Tracie A Mafile’o, Lester Asugeni, William John H McBride

**Affiliations:** 1 Pacific Adventist University, Private Mail Bag, Boroko, National Capital District 111, Port Moresby, Papua New Guinea; 2 School of Medicine and Dentistry, James Cook University, Cairns Campus, PO Box 6811, Cairns 4870, QLD, Australia

**Keywords:** Culture, Faith-based, HIV prevention, Male circumcision, Research process, Pacific Islander, Papua New Guinea

## Abstract

**Background:**

Undertaking HIV research in the culturally diverse Pacific nation of Papua New Guinea (PNG) requires careful consideration of social, cultural and religious beliefs and practices. Here, we share a detailed description of culturally informed research processes and lessons learned from the first ever study undertaken on male circumcision for HIV prevention at a faith-based university in PNG.

**Methods:**

Male and female staff and students at Pacific Adventist University were invited to complete an anonymous self-administered questionnaire, and/or participate in a semi-structured interview or focus group discussion. Male participants were invited for clinical examination. Results were collated and disseminated to the university community in gender segregated sessions. The study deliberately partnered with student leaders and centralised social, cultural, and religious paradigms. Student leaders were interviewed about their experience of partnering in sensitive health research.

**Results:**

The student leaders reported that pre-existing relationships, cultural ties, gendered sensitivity and regular communication reinforced trust between researchers, student leaders and participants, and helped the success of the study. The amount of time, complex logistics and social and cultural relationships between single and married staff and students were highlighted as challenges.

**Conclusions:**

Partnering with regional student leaders to plan and implement the study gave a legitimate and immediate mechanism for involving PNG staff and students in this sensitive health research. Gendered research processes utilised established social and cultural structures and ensured the safety of participants; all of these factors contributed to the acceptability of the study. Capacity was strengthened in PNG and Australian researchers to undertake sensitive HIV research in PNG. The study demonstrated that it is possible to conduct sensitive sexual health research at a faith-based university in PNG.

## Background

Undertaking sensitive HIV research in the culturally and religiously diverse Pacific nation of Papua New Guinea (PNG) requires careful consideration of myriad cultural, social and religious beliefs and practices [1-4]. Investigating new ways of preventing and treating HIV is vitally important given that 90% of all HIV infections in Oceania are in PNG. In 2012, the national adult HIV prevalence was 0.79%, with 31,421 people living with HIV [5]. HIV prevalence varies across the more than 800 language groups in PNG and it is primarily transmitted via heterosexual intercourse. Risk of infection is exacerbated by high levels of untreated sexually transmitted infections (STIs), unprotected sex with multiple partners, the young age at which women experience sexual debut, and low circumcision rates [5-7]. Gender inequalities and widespread physical and sexual abuse increase the vulnerability of women to HIV [4,8,9]. Weak health care delivery systems and limited surveillance (although dramatically expanded in recent years) also contribute to the partial ability to detect and treat STIs including HIV [7,10-14]. The Abstinence, Be Faithful and use a Condom prevention methodology is challenged by supply chain problems, anti-condom sentiments by some religious groups, and limited incorporation of cultural concepts of sex in HIV prevention [3,15]. After almost two decades of research on the protective effect of male circumcision on HIV transmission, the World Health Organization now recommend that male circumcision be included in comprehensive HIV prevention packages in populations with a generalised HIV epidemic, predominantly heterosexual transmission and low male circumcision rates [16-24]. Given the extreme diversity of the population, local research evidence is essential to inform any potential intervention.

### Male circumcision in Papua New Guinea

A wide range of surgical, traditional and contemporary male foreskin cutting and genital modification practices have been documented across PNG in both historical and contemporary studies. These include longitudinal and circumferential cuts of the foreskin, insertion of foreign objects under the penile skin and injection of potions and oils [3,25-31]. In 2006, it was estimated from health system data that less than 20% of men in PNG were circumcised [32,33]. However, this may not have accounted for the numerous forms of foreskin cutting ocurring outside the formal health system in villages and towns across the country [3,34]. Given the potential of male circumcision to reduce the heterosexual transmission of HIV, researchers at Pacific Adventist University (PAU, PNG) and James Cook University (JCU, Australia) established a pilot study to inform a larger collaborative study to investigate the acceptability of male circumcision for HIV prevention in PNG.

### Research partnerships that incorporate science, culture and religion

PAU is a small faith-based university owned and operated by the Seventh-day Adventist (SDA) church. It is situated 20 km from Port Moresby, the capital of PNG. PAU staff and students come from many Pacific Island Countries and Territories, although the majority are from PNG. Most staff and students live on campus and there is an expectation that staff and students follow SDA religious, spiritual and behavioural traditions while on campus.

In recent years, PAU has strengthened its research activities and consequently enhanced its ability to create new knowledge and provide high quality tertiary education relevant to the Pacific context. Creating new knowledge through this study meant first extending our understanding of research about sensitive health topics from a Melanesian perspective.

We started from the standpoint that cultural practices and taboos in Melanesia vary greatly from place to place; nevertheless, there are common practices that bind across the spirit of Melanesia [35]. When one lives and works in a Melanesian society one must make meaning of everything in terms of culture, religion and one’s social status.

Human relationships are central to Melanesian societies. For the majority of Melanesians there is a very clear knowledge and understanding of family, tribal, or clan lineage. One is either a brother, an uncle, grandfather, niece, nephew or a relative, directly or through marriage. When travelling one always carries the family and tribal identity with them. This can be both positive and negative. The connection to family and tribe provides social support, but can also mean obligations such as involvement in tribal fighting or financial contributions to compensation payments, which individuals may have limited influence over.

Exchange, giving and taking, is an integral part of Melanesian societies. People work and assist others in the Melanesian spirit of caring for others in the group. People expect to be recognised for their generosity and helpfulness, and to be appreciated. In a village when one asks for food, firewood, water or even to build a house or make a garden, everyone in the community comes to assist.

Respect for the elders and chiefs is of paramount importance in Melanesian societies. Talking back in an insulting manner is rude and unacceptable behaviour for elders and chiefs. As a way of respect people will follow advice or comply with requests. Co-operation and mutual support is also a feature of Melanesian society especially in times of need and crises. People come together to resolve problems and issues in villages and communities when inevitable tensions arise with a system of verbal and material exchange to make peace. All of the above form part of the daily living experience as Melanesians and for people living, working and researching in Melanesia.

There are numerous traditional practices that have the principles or rules of do’s and don’ts (taboos and secrets) across PNG. These are of paramount importance in each of the almost 800 Melanesian societies that make up PNG’s broader society. People from within each society, and from other societies, generally accept and live together and respect these taboos and secrets. When there is conflict and tribal fighting, it is usually resolved using a system of compensation to return the groups or societies to an equilibrium.

In PNG, as in most Pacific Island countries and territories spirituality and religion is an integral part of individual and collective identity. As Butt and Eves highlight, in Melanesia Christianity has a remarkable influence over people including its association with power: “*Christianity is the most pervasive and influential religion in the Pacific, a vital part of the framework though which most people make sense of the world*” [36]. To most Pacific Islanders, spirituality is “sacred” and is broadly understood as that which is set apart from the ordinary and worthy of reverence and respect. Spirituality can be sought not only through traditional organized cultural and ancestral practices, but also through ecological (environmental) spirituality. For this reason, one cannot discuss sexuality or other related topics openly in church buildings, as these are sacred and such topics are perceived as too taboo or 'sinful’ to be discussed in these premises.

It is from this perspective that we, as Pacific Islander and Australian researchers, approached the study of what can be learned about male circumcision with a cohort of staff and students at a Papua New Guinea university. In this paper, we report upon the process of undertaking the study at PAU and share lessons we have learnt as Pacific Islander and Australian researchers undertaking sexual health research in PNG.

### Process

#### Establishing the study

The purpose of this pilot study was to 'test run’ the research design, including tools under the same conditions in which the larger multi-site study was to be conducted [37]. In 2009, PAU academics submitted an application to PNG National AIDS Council Secretariat for funding and ethics review. A number of researchers from JCU (Cairns, Australia) joined the study, all with extensive health service and health research experience in Melanesia [38-46].

The objectives of the study were to i) describe and categorise male circumcision and genital modification in men; ii) examine how social, cultural and religious understandings and practices of male circumcision influence the acceptability of male circumcision in HIV prevention strategies; and iii) develop research capacity and inform the multi-site collaborative study on acceptability and feasibility of male circumcision as an intervention to reduce HIV transmission in PNG. Additionally, specific questions of interest included: Will people talk about circumcision? Will people talk about sex? Will men undergo physical examination and/or photography? Are the questionnaires, interviews and focus group discussions able to collect relevant information from participants? What is the prevalence of male circumcision in the university community?

The study was overseen by the Director of Research and Postgraduate Studies and supported by JCU researchers. Both male and female researchers were intentionally involved in the leadership of the study. The development of the application, drafting questionnaires, analysis and reporting data were led by PAU researchers, supported by JCU researchers. Ethics approval for the study was granted by the PAU Ethics Committee and the PNG National AIDS Council Research Advisory Committee.

During initial planning, the PAU research team invited senior PAU staff to join the study Steering Committee to provide critical advice on how to approach this study population with culturally and religiously sensitive questions. The steering committee comprised of Deans of the five PAU Schools and the Heads of Support Services. The university leaders on the steering committee were from various countries: PNG, Tonga, Solomon Islands, Samoa, Fiji, South Africa, Australia and New Zealand. The conservative nature of the church-run university, the small size of the campus, the limited amount of research undertaken by the university at that time and the contentious nature of the study meant that support of university leaders was critical if the study were to succeed. Support for the study was forthcoming with advice that the research team partner with student leaders from each of the four PNG regions.

### Student leaders as research partners

Geographically, PNG is grouped into four regions made up of collections of neighbouring provinces: Southern, Momase, Niugini Islands and Highlands regions. Although there is diversity within each regional group there are many shared social and cultural practices and worldviews. Staff and students from across all regions of PNG come to work or study at the university. The university therefore recognises these regional groups as the primary channel of networking within the campus amongst the PNG staff and students. The student association, and student activities, including social, cultural, academic and religious events are primarily organised according to these regional groups. Here forthwith, we will refer to these leaders as regional leaders within this broader social, cultural, institutional and religious context.

The four regional leaders (3 male and 1 female) were requested to partner with the research team and each leader enthusiastically embraced the opportunity. Regional leaders partnered with research leaders to disseminate information to participants in their regional groups; feedback information to the researchers from potential participants; distribute and collect questionnaires; and help recruit participants and organise one-on-one interviews and focus group discussions. Regional leaders gave feedback to the research team on key lessons learnt from the study to inform the larger multi-site study. Leaders worked with their assistant leaders who were of the opposite sex, making it possible to disseminate gender specific information and questionnaires designed separately for male and female participants. Each regional group leader received mobile phone credit to enable communication between leaders and members of their group as well as the research team. Email was also used by the student leaders to communicate. At the completion of the study a donation was made proportional to the size of the regional group. Between PGK40–200 (USD 20–100) was forwarded to each regional group’s fundraising account to acknowledge the contribution of the group and enable each cultural group to benefit collectively from participation in the study.

### Promotion of the study

The study was introduced to PAU staff and students through the PAU newsletter *Harina* and via the weekly Student Assembly. This Assembly provides a forum for staff and students to present and/or hear about academic topics and issues relevant to the university body. Regional leaders also disseminated information during their regular group meetings. Informal word of mouth was an important promotion tool. The presence of two JCU researchers living on the PAU campus while the study was being promoted also raised the profile of the study and highlighted opportunities for staff and students to participate.

### Data collection

Data was collected during April 2010 using i) separate male and female self-administered questionnaires; ii) semi-structured interviews; and iii) focus groups discussions. Males were invited for voluntary optional clinical examination on the last page of the male questionnaire. Participants were PNG staff and students 18 years old or above. All participants were provided with information sheets clearly explaining the study and the expected use of data. Consent forms were completed by participants prior to individual interviews, focus group discussion and clinical examination. Consent forms were not provided to participants completing anonymous self-administered questionnaires, with informed consent assumed with the voluntary return of the completed questionnaire. The anonymous questionnaire collected data on demographics, knowledge of HIV and STI, sexual behaviours and attitudes and practices of male circumcision. Completed questionnaires were returned by 37 of 46 females (80%) and 59 of 137 males (43%). The questionnaires were completed to a high standard with many participants writing lengthy comments to open ended questions.

A purposive sampling technique was used to identify potential study participants with knowledge/experience about male circumcision and foreskin cutting for individual interview and focus groups with regional leaders. Eight semi-structured interviews were conducted, four male and four female, in mutually agreed and neutral locations on the PAU campus. Female researchers interviewed women and male researchers interviewed men. Three focus group discussion groups were facilitated (single female, single male, married male) involving 8 women and 13 men. Consistent with common ways of social grouping and delivering services in PNG and the Pacific, separate focus group discussions were conducted for single and married participants. Recorded data from approximately 10 hours of interviews and group discussions described a great diversity of foreskin cutting styles and practices across PNG.

Male participants were invited to travel to Port Moresby General Hospital (about 20 km from PAU campus) for voluntary clinical examination and an anonymous photography of their genitals to compare to self-reported circumcision status from questionnaires. Males removed the last page of the male questionnaire which contained information about the voluntary clinical examination, directions to the hospital and the questionnaire number. This page was then given to the medical officer at presentation at the hospital. The medical officer recorded the clinical foreskin cutting classification, and attached the photos, noting the unique identifying code number for the participant. This information was returned to the researchers to enable the clinical examination data be matched with the questionnaire data. Thirteen male participants presented for clinical examination, although 4 of these had not returned a completed questionnaire. Because participation was anonymous and only participant numbers were recorded, there was no way to trace these individuals to encourage them to submit their questionnaire. Each male participant who presented for clinical examination was given PGK20 (USD10) to assist with transport to and from the hospital and a meal while off campus.

Confidentiality and anonymity were of paramount importance given the sensitive nature of the study which recorded data on sexual behaviour and foreskin cutting practices, particularly in the context of conducting it at a faith-based university. Following SDA behavioural expectations, non-married PAU students who are found to have had sex on campus are expelled from their studies and student accommodation. Thus, from a student perspective, there were potentially high costs of participating in the study. Specific steps taken by researchers to ensure confidentiality and anonymity included questionnaires being distributed by regional group leaders who were also students; female regional leaders distributed to females and male regional leaders distributed to males; questionnaires were numbered, but no names recorded on the questionnaires; questionnaires were returned in a sealed envelope to a sealed box in locations across the university campus including library and in student dormitories, or returned to regional group leaders who placed the already sealed envelopes into the sealed boxes; and anonymous questionnaires were stored in a secure office according to ethics requirements and data stored securely on password protected computers. Semi-structured interviews and focus group discussions were always conducted in private locations on campus and students were assured that frank discussion about sexual and circumcision practices would not result in suspension or expulsion. Students were also assured that data would be kept confidential with no names able to be linked with questionnaires or photographs or used in reporting.

### Student leader reflections and inter-university collaboration

Once data collection was complete, a group debrief session was facilitated to discuss the research process with the four regional leaders. Key lessons for the study were systematically recorded and categorised as barriers or enablers.

Additionally, an inter-university contribution was made by one regional leader. The Niugini Islands regional group leader, a female, travelled with the PAU/JCU research team to Divine Word University (DWU), Madang Campus. There, she described the processes that had been employed at PAU and the key lessons learned in a student-to-student dialogue which contributed to partnerships with student leaders at DWU involved in the multi-site study later that year.

Reflections from regional leaders on the study process during debriefing sessions were categorised as enablers and barriers. Enablers were i) regular communication between researchers and leaders: meetings, phone calls, informal contact; ii) provision of mobile phone credit to ensure good communication between researchers and regional leaders and also regional leaders with research participants; iii) email used as an effective communication tool between researchers and regional leaders; iv) pre-existing relationships and cultural ties between researchers and regional leaders meant levels of trust were high prior to the commencement of the research; v) trust was also high between researchers and participants because of the approach taken to disseminate information to males and females separately. The male regional leaders worked closely with male participants and female regional leaders with female participants; vi) regional leaders regularly, and often spontaneously, provided feedback to researchers on the progress of the data collection because of enthusiasm for the project; vii) regional leaders learnt more about HIV as a result of partnership in the study: this was good personally and for sharing with friends and family; viii) the monetary gifts of thanks given to the regional groups (rather than to individuals) contributed to fundraising for important cultural events at the university; ix) regional leaders were senior student leaders and appreciated the opportunity to be partners in research activities. Regional leaders reported increased motivation to participate more fully in compulsory research subjects undertaken as part of their university courses; x) one regional leader inquired about employment with the research team after their graduation.

Barriers were i) the large amount of time it took to organise participants for the study, especially over weekends when regional leaders were not always available on campus; ii) uncertainty about the collection of the questionnaires: some participants did not want to put sealed envelopes containing completed questionnaires in the boxes in 'public places’ like the university library. This improved when students had options to return questionnaires in less public areas such as into boxes in the male and female dormitories; iii) clinical examination at a hospital 20 km away meant a large commitment of time by male participants. Some indicated they were happy to participate, but were not willing to devote the time in travel to and from the hospital. Male participants suggested future clinical examinations be conducted on the PAU campus; iv) most married students and staff did not participate because all regional leaders were single students. This resulted in lower participation from married staff and students in the study.

In addition, male regional leaders forwarded a request to the research team that a routine medical male circumcision service be provided for uncircumcised PAU students at the PAU medical centre. Male circumcision is not a procedure offered at the PAU medical centre (nor many other medical centres) and therefore many students reported having had the procedure done by a friend or family member away from any medical system or medical supervision.

## Discussion

This is the first study conducted on the beliefs, attitudes and practices of male circumcision at a faith-based university in PNG. It therefore provides not only vital data on male circumcision and other forms of foreskin cutting but also enables reflection on the participatory research processes underpinned by science, faith and culture. The diversity of foreskin cutting styles and practices (including male circumcision) documented in the study informed the development of a more sophisticated and culturally nuanced questionnaire for use in the multi-site study that followed [29,47,48]. It was significant that the PAU researchers on the study were influential, well-respected senior staff, including the Director of Research at the university. This social and academic positioning, along with the personal and cultural nature of relationships between staff and students at this small campus, contributed to high levels of trust for what could have been a contentious and threatening research topic. More importantly, our experience was that the cultural binds between students and researchers superseded formal positions held as university academics. Given the relative religious homogeneity of staff and students (100% of PAU staff are SDA and 70–80% of the student population are SDA), cultural (and related linguistic) bonds between students and researchers appeared to take precedence over religious and behavioural expectations; this enabled open and frank discussion. The non-judgemental approach to issues of sex and foreskin cutting was a central principle enacted by PAU researchers and also enabled open discussion with students. The grouping of research participants in regional/cultural groups engendered a sense of safety and security for participants, despite reporting sexual activities and foreskin cutting procedures that may have breached the SDA church and PAU’s behavioural expectations.

Students were assured in the study Information Sheet and at the beginning of the interviews and focus group discussions that their participation in the study would not result in suspension or expulsion from the university despite the potential for expulsion that non-married students live with if they are sexually active on the PAU campus. This commitment by researchers, the PAU Ethics Committee and the PAU university administration team to support the study, along with the anonymity provided for those participating in the structured questionnaire, enabled a safe environment for men and women to participate without fear of personal, professional or administrative consequence. The advice provided by the PAU steering committee to partner with the regional groups also provided confidence for the research team to undertake this potentially contentious study.

Results collected in the study indicate that indeed staff and students at a faith-based university in PNG will talk about male circumcision, their knowledge of HIV and their sexual histories, and that men will undergo clinical examination and photography. Comprehensive data is able to be collected using questionnaires, individual interviews and focus group discussion if conducted in a way that acknowledges and works with existing social, cultural, religious and administrative structures. The responses from participants assisted in developing a more detailed and nuanced questionnaire for the multi-site study which included specific sections for men with different forms of foreskin cutting (longitudinal cuts and circumferential cuts) to enable a more detailed understanding of foreskin cutting in PNG [29].

This study also enabled us to develop research capacity to undertake similar studies in PNG. However, our experience in this and other studies in the Pacific shows that research must be undertaken using de-colonising approaches [49-51]. Developing research capacity has been experienced by some in hegemonic ways which extend the power of the privileged 'research capacity builders’ from more resourced countries over those intended to 'benefit’ from the capacity building [52]. Our experience is that for capacity building undertaken in a de-colonising way, with power shared between researchers and other research partners (such as student leaders), the process can be beneficial for all involved in the research [42]. Within this context, the capacity to undertake sensitive health research was strengthened in PAU researchers, regional leaders and JCU researchers. For those team members starting with limited research experience, this study helped to further develop their research skills. Experience was gained by undertaking the various research activities: reviewing literature; formulating research questionnaires; writing proposals and ethics applications; collecting and analysing data; and presenting preliminary findings to various sites locally and internationally. An extensive network of support was available both from our own university (PAU) and JCU, our collaborative research partner. Having international researchers living on the PAU campus during the pilot study was important as it provided immediate support when required.

For those team members with more research experience, our capacity to undertake HIV research in PNG was enhanced by the opportunity to live and/or work closely with PAU colleagues and more fully understand the cultural and spiritual context when undertaking sexual health research in PNG. We have discovered alternative ways of conducting both quantitative and qualitative research. The lessons learnt from the study were of great benefit to all of us in informing the multi-site study that followed [29,53].

## Conclusions

This study aimed to describe and categorise male circumcision and penile modification, examine social, cultural and religious understandings of male circumcision, and to strengthen research capacity at PAU. All of these aims were achieved through mutual and respectful partnerships between international research institutions as well as established leadership structures within the PAU student body. Staff and students at a faith-based university in PNG will participate in sensitive sexual health research when supported by regional leaders in the context of trusting relationships with researchers and ethical research practices. Men and women will report their attitudes and practices about male circumcision and knowledge of HIV, including related sexual histories.

Having regional leaders is an effective way to establish and maintain linkages with student and staff populations at a PNG university. In this instance, these bonds have proved stronger than other forms of collective identity such as religious/denominational expectations or professional/academic position. These bonds directly influenced the willingness and enthusiasm to participate and disclose information for the study. Practical support to enable regional leaders to undertake their tasks within the research partnership (such as mobile phone credit) proved to enable research in a context of limited financial resources. Time, complex logistics and social and cultural relationships between single and married staff and students were identified as challenges for further study. Capacity has been strengthened in all researchers and institutions involved in this sensitive sexual health research.

## Abbreviations

JCU: James Cook University; PAU: Pacific Adventist University; PNG: Papua New Guinea; SDA: Seventh-day Adventist; STIs: Sexually transmitted infections.

## Competing interests

The authors declare there they have no competing interests.

## Authors’ contributions

RT: Designed the pilot study, led data collection and analysis and contributed to the manuscript; DM: contributed to the design of the pilot study, supported data collection and analysis while living at PAU and revised the manuscript; MRM: contributed to the design of the pilot study, supported data collection and analysis while living at PAU and drafted the manuscript; TM: co-designed the pilot study, supported data collection and analysis and contributed to the manuscript; LA: co-led data collection, contributed to data analysis and revised the manuscript; WHJM: conceptualised the male circumcision project and arranged for initial meeting of researchers, provided advice throughout the pilot and revised the manuscript. All authors read and approved the final manuscript.
